# Developing custom computer vision models with Njobvu‐AI: A collaborative, user‐friendly platform for ecological research

**DOI:** 10.1002/eap.70096

**Published:** 2025-09-11

**Authors:** Cara L. Appel, Ashwin Subramanian, Jonathan S. Koning, Marnet Ngosi, Christopher M. Sullivan, Taal Levi, Damon B. Lesmeister

**Affiliations:** ^1^ Department of Fisheries, Wildlife, and Conservation Sciences Oregon State University Corvallis Oregon USA; ^2^ Pacific Northwest Research Station USDA Forest Service Corvallis Oregon USA; ^3^ Oak Ridge Institute for Science and Education Oak Ridge Tennessee USA; ^4^ Center for Quantitative Life Sciences Oregon State University Corvallis Oregon USA; ^5^ Nkhotakota Wildlife Reserve Nkhotakota Malawi; ^6^ College of Earth, Ocean, and Atmospheric Sciences Oregon State University Corvallis Oregon USA

**Keywords:** Africa, artificial intelligence, camera traps, machine learning, Malawi, software, wildlife monitoring

## Abstract

Computer vision models show great promise for assisting researchers with rapid processing of ecological data from many sources, including images from camera traps. Access to user‐friendly workflows offering collaborative features, remote and local access, and data control will enable greater adoption of computer vision models and accelerate the time between data collection and analysis for many conservation and research programs. We present Njobvu‐AI, a no‐code tool for multiuser image labeling, model training, image classification, and review. Using this tool, we demonstrate training and deploying a YOLO multiclass detector model using a modest dataset of 33,664 camera trap images of 37 animal species from Nkhotakota Wildlife Reserve, Malawi. We then applied our model to an independent dataset and evaluated its performance in terms of filtering empty images, species classification, species richness, and per‐image animal counts. Our model filtered over 3 million empty images and had similar sensitivity but lower specificity than the MegaDetector model at differentiating empty images from those with animals. Classification performance was high for species with >1000 training images (average precision, recall, and *F*1 >0.70) and moderate overall (macro‐averaged precision = 0.64, recall = 0.76, *F*1 = 0.63). Site‐level species richness using predicted detections with and without manual review were highly concordant, especially when a score threshold of 0.95 was applied ($R^{2}$ = 0.91). Counts of animals per image were predicted accurately for many species but underestimated by up to 22% for those in large groups. This workflow represents an option for researchers to implement custom computer vision models for even modest‐sized ecological datasets in an all‐in‐one, collaborative, no‐code platform.

## INTRODUCTION

Passive monitoring methods like camera traps have revolutionized data collection for biodiversity research and monitoring (Oliver et al., 2023; Steenweg et al., 2017). Camera traps enable data collection on wildlife species occurrences, behavior, and population status at broader spatial and temporal scales than direct observations or intensive methods like animal‐borne movement trackers (Burton et al., 2015; Wearn & Glover‐Kapfer, 2019). However, processing and analyzing the large volumes of data collected by camera traps is labor‐intensive and can delay the timely analysis and reporting of results, thus reducing the value of camera traps for research and monitoring programs. Fortunately, new computational methods using machine learning show great promise for improving data processing from camera traps, autonomous recording units, and other remote sensors and are increasingly being applied to ecological data (Pichler & Hartig, 2023; Tosa et al., 2021; Tuia et al., 2022).

Computer vision, a type of machine learning that performs various image processing tasks, is particularly useful for filtering, detecting, classifying, and individually identifying animals or their vocalizations in images or spectrograms from acoustic recordings (Norouzzadeh et al., 2017; Ruff et al., 2020; Salamon & Bello, 2017; Schneider et al., 2019; Tabak et al., 2019). For camera trap projects, filtering empty images is a common bottleneck, and previous studies have focused on leveraging computer vision models to reduce human review time by separating images with animals from empty images or images with people (Fennell et al., 2022; Meng et al., 2023; Norouzzadeh et al., 2021; Willi et al., 2019; Yang et al., 2021). Foundational detector models such as MegaDetector are now widely used to filter images with animals from empty images caused by camera false triggers (Beery et al., 2019). Beyond detection, other recent tools have made the application of species classification models more accessible to ecologists—for example, the platforms Wildlife Insights (Ahumada et al., 2020) and Agouti (Casaer et al., 2019) offer cloud storage, data management, and multiuser access as well as global or regional species classification models. Other desktop‐based applications allow users to process their own images using a variety of regional species classifiers—for example, DeepFaune (Rigoudy et al., 2023), AddaxAI (formerly EcoAssist; van Lunteren, 2023), Mbaza (Whytock et al., 2021), and Trapper (Bubnicki et al., 2016). Applications like these have the potential to increase the impact of wildlife monitoring programs through more efficient data processing, and many practitioners are seeking to incorporate computer vision methods into even small‐scale projects (e.g., Duggan et al., 2021).

Despite the advantages of computer vision, the lack of generalizability is a common problem for these models and for camera trap studies in particular (Beery et al., 2018; Koh et al., 2021). Species classification models often show poor performance when applied to data from camera locations unseen during training (Beery et al., 2018; Schneider et al., 2020). Images from new locations or time periods (“out of distribution” data) often have different backgrounds, camera angles, image quality, lighting, and other features that lead to less accurate predictions (Beery et al., 2018; Norman et al., 2023; Schneider et al., 2020). The distribution of species classes in new image datasets may also vary considerably from species in the training images for a given model. For application to new datasets, users are likely to observe lower performance compared to models trained with more similar distribution between training and application datasets. Therefore, training custom, project‐specific models may be beneficial for many ecological purposes.

Computer vision models for camera trap images have often relied on large, labeled datasets for training, like the Snapshot Serengeti dataset (Gomez Villa et al., 2017; Norouzzadeh et al., 2021; Swanson et al., 2015; Tabak et al., 2019; Willi et al., 2019). However, custom models could potentially be used as a preliminary step in data processing for monitoring or research programs that lack existing, labeled datasets, in a way that prioritizes timely reporting and analysis while providing a pathway for future model improvement. As an example, Ruff et al. (2020) annotated a dataset of approximately 71,000 spectrogram images from audio‐recordings of six forest owl species and trained an initial model, which was then applied to larger datasets containing millions of images. Reviewing model predictions served to curate additional annotated images for retraining to improve performance and expand the scope of the model to include 37 total classes while producing vetted datasets for ecological analysis (Ruff et al., 2021, 2023). The iterative nature of this process means that initial models can be both imperfect and useful, although detection models should meet precision, recall, and sensitivity standards to be most useful when applied to a larger dataset. In evaluating model performance, researchers may also prioritize being able to make ecologically meaningful inference directly from preliminary model predictions without extensive review, in which case the accuracy of metrics like species richness (the number of species detected at a site), per‐image animal counts, or occupancy rates will be important (e.g., Lonsinger et al., 2024; Whytock et al., 2021).

Broad application of custom computer vision models requires computing resources and skills outside the scope of many ecological research groups, including expertise in computer programming and software engineering (Cole et al., 2023). Existing tools provide robust options for annotating data—for example, LabelMe (Russell et al., 2008), Label Studio (Tkachenko et al., 2022), and CVAT (CVAT.ai Corporation, 2023), which even produce outputs in standard formats for training machine learning algorithms. Model training is then commonly implemented separately, using command‐line tools to access Python libraries like TensorFlow (Abadi et al., 2015), PyTorch (Ansel et al., 2024), and Ultralytics (Jocher et al., 2023), although code repositories specific to training models for camera trap data are now available in the R programming language (Böhner et al., 2023; Schneider et al., 2020; Tabak et al., 2020), which is more commonly used by ecologists. Such approaches are valuable for encouraging users to learn the nuances of model training and are likely to promote greater intuition about machine learning among ecologists, as reliance on hands‐off tools offers fewer opportunities for users to customize their workflows and understand the process more deeply (Cole et al., 2023). However, extensive programming training is not available or practical for many ecologists and conservation practitioners, and no‐code options are a valuable step for quickly implementing computer vision workflows and provide an entry point for building capacity. The recent development of tools offering graphical user interface options to train custom models, including AIDE (Kellenberger et al., 2020) and Project Zamba (https://zamba.drivendata.org/), demonstrates a clear need for practical tools to make the development of custom models more accessible for conservation programs seeking to use computer vision methods to increase their impact.

**FIGURE 1:**
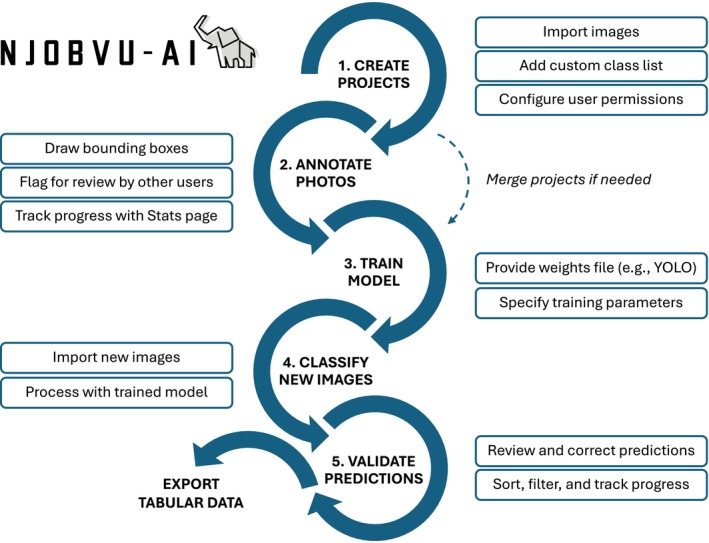
Workflow for using Njobvu‐AI software to implement computer vision models with ecological data. Logo for Njobvu‐AI was created by Cara L. Appel using BrandCrowd (https://www.brandcrowd.com).

Here, we present a comprehensive software tool, Njobvu‐AI, which provides collaborative, user‐friendly features for developing custom models (Figure 1). Njobvu‐AI shares many features with existing labeling tools—the ability to work locally or on a remote server, multiuser capability, and quality‐control measures—and offers the added feature of implementing model training and inference through a browser‐based graphical user interface. The name Njobvu‐AI (pronounced N‐joh‐voo A I), incorporating the word for “elephant” in the Chichewa language of Malawi, references similarities between artificial intelligence models and the powerful memory of African bush elephants (*Loxodonta africana*).

Using Njobvu‐AI, we demonstrate the development of a custom computer vision model using camera trap images from a wildlife restoration project in Nkhotakota Wildlife Reserve, Malawi. With over 200 camera stations collecting millions of images per year, the monitoring program requires efficient data processing workflows to produce population assessments and meet other reporting goals. We annotated a modest dataset of approximately 33,000 camera trap images from the early years of the monitoring program using bounding‐box labels. We used this dataset to train a YOLO multiclass detector model for classifying animals from 37 species classes, then applied the model to a newer dataset of approximately 3.5 million images and performed a collaborative review of model predictions. To prioritize the ecological relevance of the data along with robust performance metrics, we evaluated our model on sensitivity with respect to filtering empty images, species classification performance, species richness comparisons from model predictions with and without manual review, and accuracy of per‐image animal counts.

## METHODS

### Software

Njobvu‐AI facilitates collaborative image labeling by allowing multiple users to share and merge projects for annotation and review while offering the ability to use centralized computing resources for model training and inference. Using the tool, users can draw bounding boxes around objects (e.g., animals) and assign them to classes corresponding to species or taxonomic group. Bounding‐box coordinates and class IDs are stored in project databases using SQLite3 and can be downloaded in various formats (e.g., YOLO, JSON, TXT, CSV, Pascal VOC). Although we focus on its application to camera trap studies, Njobvu‐AI is compatible with a variety of image and video inputs and can be used for other applications from aerial imagery to microscopy.

Njobvu‐AI leverages open‐source neural network programs to enable custom model training. Wrapper scripts support training multiclass object detector models using YOLOv4 architecture in the Darknet framework (Bochkovskiy et al., 2020; Redmon, 2016) and a wider variety of classification models using custom scripts for TensorFlow (Abadi et al., 2015), with capability for newer model architectures currently in development (e.g., YOLO11 with Ultralytics; Jocher et al., 2023). To train using an annotated dataset, users provide a base model weights file and specify parameters to be used for model training.

Once models are trained (or using a pretrained model), users can perform image classification on new sets of images and review predictions to evaluate model performance, generate new training data, and confirm animal detections for ecological analysis. The tool's review mode includes filters to sort and display images by predicted class and confidence score, as well as an automatic “Needs Review” flag to track which images have not yet been viewed. Each project has a statistics page to display the current review progress and number of predictions by species class. When reviewing predictions is completed, users can download tabular data files of corrected model predictions for further analysis.

Njobvu‐AI is supported on multiple operating systems including Windows, Mac, Linux, x86, and IBM Power. Functionality for cloud server access is enabled by Node.js so that remote machines can access the tool through web browsers with no additional software installation. Software dependencies for the host machine, or to use the tool locally, include Node.js (for all features) and Darknet (Bochkovskiy et al., 2020; Redmon, 2016) or TensorFlow (Abadi et al., 2015) for model training and image classification. Use of a graphics processing unit (GPU) is required for model training and recommended for image classification. Source code and documentation are available at https://github.com/sullichrosu/Njobvu-AI and are further detailed in Koning et al. (2023).

### Study system

**FIGURE 2:**
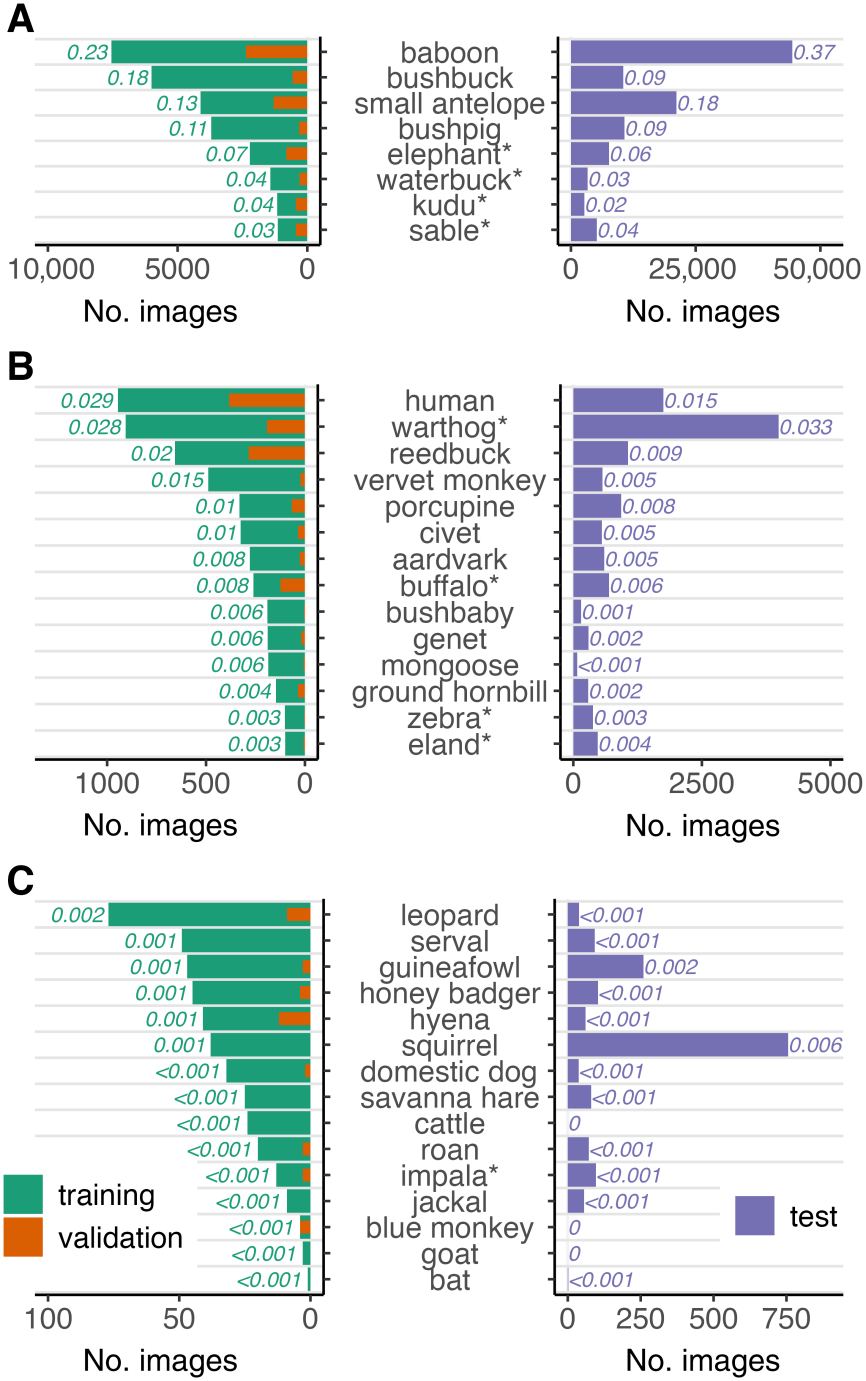
Distribution of species classes in training (green), validation (orange), and test (purple) data splits for a multiclass detector model to identify wildlife species in camera trap images from Nkhotakota Wildlife Reserve, Malawi. Labels next to the bars show the proportion of each split made up by each class (see Appendix S1 for validation dataset). Note that *x*‐axis limits differ by an order of magnitude; for visibility, classes are plotted separately by the number of images in the training dataset: (A) ≥ 1000, (b) between 100 and 1000, and (C) < 100. Asterisks (*) denote species that were part of the translocation effort.

We used wildlife monitoring data from Nkhotakota Wildlife Reserve, an 1800‐km^2^ protected area in central Malawi that operates under a partnership between the Malawi Department of National Parks and Wildlife (DNPW) and African Parks. In 2016, 2017, and 2022, large translocation efforts were undertaken to restore wildlife populations, involving the release of over 2000 individuals of nine species, including elephants and other large mammals (Figure 2). In collaboration with the USDA Forest Service, reserve staff have implemented a multispecies monitoring program using camera traps to survey the distribution and abundance of the reintroduced and extant wildlife populations.

Nkhotakota Wildlife Reserve is primarily composed of miombo woodlands (dominated by *Brachystegia* spp.) with a small patch (0.44 km^2^) of Afromontane forest. Elevation ranges from 500 m near the shores of Lake Malawi to 1637 m at the highest peak, Chipata Mountain. Two major rivers (Bua River, Kaombe River) bisect the reserve, and a third (Dwangwa River) forms the northern reserve boundary.

### Data collection

Images were collected in Nkhotakota Wildlife Reserve using motion‐activated camera traps from November 2018 to October 2023. The monitoring design is based on a grid of 5‐km^2^ hexagonal cells, with 70 cells randomly selected for monitoring. Each of these was surveyed with three cameras (Bushnell Trophy Cam HD Aggressor No‐Glow, Overland Park, Kansas, USA) spaced approximately 1.4 km apart in a triangle design. Cameras were installed on trees at a height of approximately 1.5 m and were programmed to capture images in bursts of one or three images with a delay of ≥1 min between triggers. Cameras were checked and serviced between one and four times during the study (median = 2) and were active for an average of 613 days (range 13–1731 days).

To compile training data, we annotated images with bounding boxes corresponding to the species class and number of animals using the Njobvu‐AI software. We identified animals to the species level when possible and higher taxonomic groups when necessary (e.g., small antelope, Sciuridae, Genetta spp.; see Appendix S1 for class descriptions). We first annotated images collected between November 2018 and September 2020, after which we trained our model. To assess preliminary performance during training, we held aside a validation dataset comprising 20% of these images, selected randomly, and used the remaining 80% for model training (Figure 2). Subsequently, we used the trained model to generate predictions on all images collected between September 2020 and November 2023 and used this dataset for model evaluation. We considered this a post hoc test set, as we did not annotate images manually but rather classified them using the trained model and then reviewed predictions for accuracy.

The test dataset constituted a temporally independent split from the training dataset, as no images from the same camera deployment periods were included in both datasets. Because of staggered camera deployments, the test data also represented a partial spatial split from the training data; of 189 total camera stations with data used here, 31 were represented in only the training set, 67 in only the test set, and 91 were represented in both.

### Model training

We used transfer learning to train a multiclass detector with 37 classes using the YOLO model architecture (Redmon et al., 2016). Transfer learning—starting from pretrained model weights rather than from a random starting point—increases training speed and performance by leveraging the feature detection abilities learned from millions of images and eliminating the need to train models from scratch (Todman et al., 2023). YOLO models perform bounding‐box detection, predicting the location (in pixel coordinates) and class identity of objects in each image, along with confidence scores from 0 to 1. Confidence scores for YOLO models are the product of two components: the likelihood that a box contains an object and the confidence in the correct class prediction.

For base weights, we used a YOLOv4 model pretrained on the Common Objects in Context (COCO) dataset (Lin et al., 2014) with 137 convolutional layers and with all but the last layers frozen for fine tuning (Bochkovskiy et al., 2020). We trained the model for 182 epochs (96,000 iterations with batch size = 64), subdivisions = 4, initial learning rate = 0.001, momentum = 0.949, and image size 608 × 608. Model training and image classification were done using NVIDIA Tesla V100‐SXM2 processors with 16 GB memory.

### Model evaluation

We evaluated our model using predictions on the test dataset comprising images collected between September 2020 and October 2023. We reviewed all images predicted to contain animals with confidence scores ≥0.25, as well as a subset of images predicted to have no animals (≥100 images per camera deployment when possible). We verified species classifications and ensured that the number of predicted bounding boxes matched the number of animals in the image, adding or removing boxes when necessary. Because we were most interested in correct species classification and count of animals in each image, we did not always modify or redraw imperfect boxes (e.g., boxes covering only part of an animal or including excessive background). Therefore, we did not consider the localization of boxes—typically assessed using the intersection over union between ground truth and predicted boxes (Szeliski, 2022)—instead focusing on species classification and animal counts at the image level, as well as filtering of empty images and implications for inference based on site‐level species richness.

#### Filtering empty images

We compared the performance of our model for filtering images without animals with the MegaDetector v5 model (Beery et al., 2019), which is now widely used to filter empty images due to false triggers. MegaDetector v5 is also based on a YOLO architecture (YOLOv5) and predicts detections of animals, people, and vehicles in camera trap images. We assessed agreement between our model (detection of animals of any class) and MegaDetector (detections of animals) as well as between both models and the confirmed detections using our test dataset. We calculated sensitivity (true positive [TP] rate) and specificity (true negative [TN] rate) of the models at score thresholds from 0.25 to 0.95 (by 0.1) as

$$\text{Sensitivity}=\frac{\mathrm{TP}}{\mathrm{TP}+\mathrm{FN}}$$

$$\text{Specificity}=\frac{\mathrm{TN}}{\mathrm{TN}+\mathrm{FP}}$$

using tallies of TPs, false positives (FPs), and false negatives (FNs) at the image level (all species classes were collapsed to “animal”). TNs were images without animals.

#### Species classification

We evaluated model performance based on the precision, recall, and unweighted *F*1 scores of species classifications at the image level. Precision describes the rate of correct classifications relative to all predicted detections of a species, and recall is the rate of TPs that were correctly predicted by the model. *F*1 is a metric that balances precision and recall. These metrics are defined as

$$\text{Precision}=\frac{\mathrm{TP}}{\mathrm{TP}+\mathrm{FP}}$$

$$\text{Recall}=\frac{\mathrm{TP}}{\mathrm{TP}+\mathrm{FN}}$$

$$F1=2\times \frac{\text{Precision}\times \text{Recall}}{\text{Precision}+\text{Recall}}$$

where TP, FP, and FN were tallied at the image level as described below. We calculated precision, recall, and *F*1 separately for each species class at various score thresholds from 0.25 to 1.0, incremented by 0.05. For example, at a threshold of 0.50, only model predictions with scores ≥0.50 were considered as detections. For images with more than one predicted animal, we used the highest confidence score among them for thresholding. These threshold‐specific metrics are useful for identifying appropriate minimum scores for review, but we also calculated threshold‐independent metrics to assess overall model performance, including average precision, average recall, and average *F*1 across all confidence score thresholds (0.25–1.0 by 0.05) for each species class. We further averaged these across classes to compute macro‐averaged precision, recall, and *F*1.

To determine TP, FP, and FN at the image level, for each species *s*, we selected images with manually confirmed presence of that species in our test dataset. We then found model prediction(s) for each of those images. If the true species was among the predicted species, we counted that image as a TP with respect to species *s*; otherwise, it was a FN (i.e., none of the predictions matched the true species). We then tallied all remaining images predicted to contain species *s* (but which were not confirmed to) as FPs. TNs were not defined for these metrics. We iterated through a sequence of thresholds *t* (0.25–1.0 by 0.05), removing predictions with scores <*t*, tallying TPs, FPs, and FNs and recalculating metrics each time.

Finally, to test whether classification performance was related to training data size, we fit linear models with average precision, recall, and *F*1 as the response variables and the number of training images per class—or the log_10_ of this value—as the predictor variable (Appendix S2). Linear models were run in R using the *lm* function (R Core Team, 2024).

#### Species richness

We calculated species richness—the number of species detected at each site—using model predictions and using verified detections as a more ecologically meaningful metric of evaluating our model's performance. We followed methods from Whytock et al. (2021) to compare estimates using linear regression, with model‐predicted species richness as the response variable and species richness calculated using manually verified detections as the predictor variable (Appendix S3). We calculated species richness both ways at score thresholds from 0.25 to 0.95 (by 0.10) and report $R^{2}$ and slope values from the linear models. Finally, to assess concordance of species richness in addition to correlation, we calculated absolute bias as the difference between model‐predicted species richness and observed species richness at each confidence score threshold.

#### Animal counts

We further assessed the performance of our model at correctly predicting counts of animals at the image level using linear regression (Appendix S4). To test which classes had differences between true and predicted counts and to estimate the additive magnitude of the differences, we modeled $\left({\text{Count}}_{\text{true}}-{\text{Count}}_{\text{predicted}}\right)$ as a function of species class. To estimate the proportional magnitude, we used $\left({\text{Count}}_{\text{predicted}}/{\text{Count}}_{\text{true}}\right)$ as the response variable instead. Finally, to estimate true counts as a function of model‐predicted counts, we modeled ${\text{Count}}_{\text{true}}$ as the response variable and an interaction term $\log\left({\text{Count}}_{\text{predicted}}\right)\times \text{Class}$ as the dependent variable, using a generalized linear model with the Poisson distribution. All models used only data for TP images—those with animals correctly classified—that had only one species present.

## RESULTS

Over 5.6 million images were collected between 2018 and 2023 in Nkhotakota Wildlife Reserve. Of the images collected prior to September 2020, we manually filtered empty images (i.e., images resulting from cameras being triggered by vegetation movement but containing no animals) and annotated the remaining images to species class. From these, we compiled a training dataset of 33,664 images, approximately 3% (*n* = 968 images) of which were empty, from 122 camera stations in 45 hexagon cells. We then used this dataset to train a YOLOv4 multiclass detector on 37 species classes (Appendix S1). Our training dataset exhibited class imbalance, with between <5 images (bat spp., blue monkey *Cercopithecus mitis*, domestic goat) and >7000 images (yellow baboon *Papio cynocephalus*) per species class (Figure 2). The validation split contained 8462 images distributed randomly among classes (Appendix S1).

### Model evaluation

**FIGURE 3:**
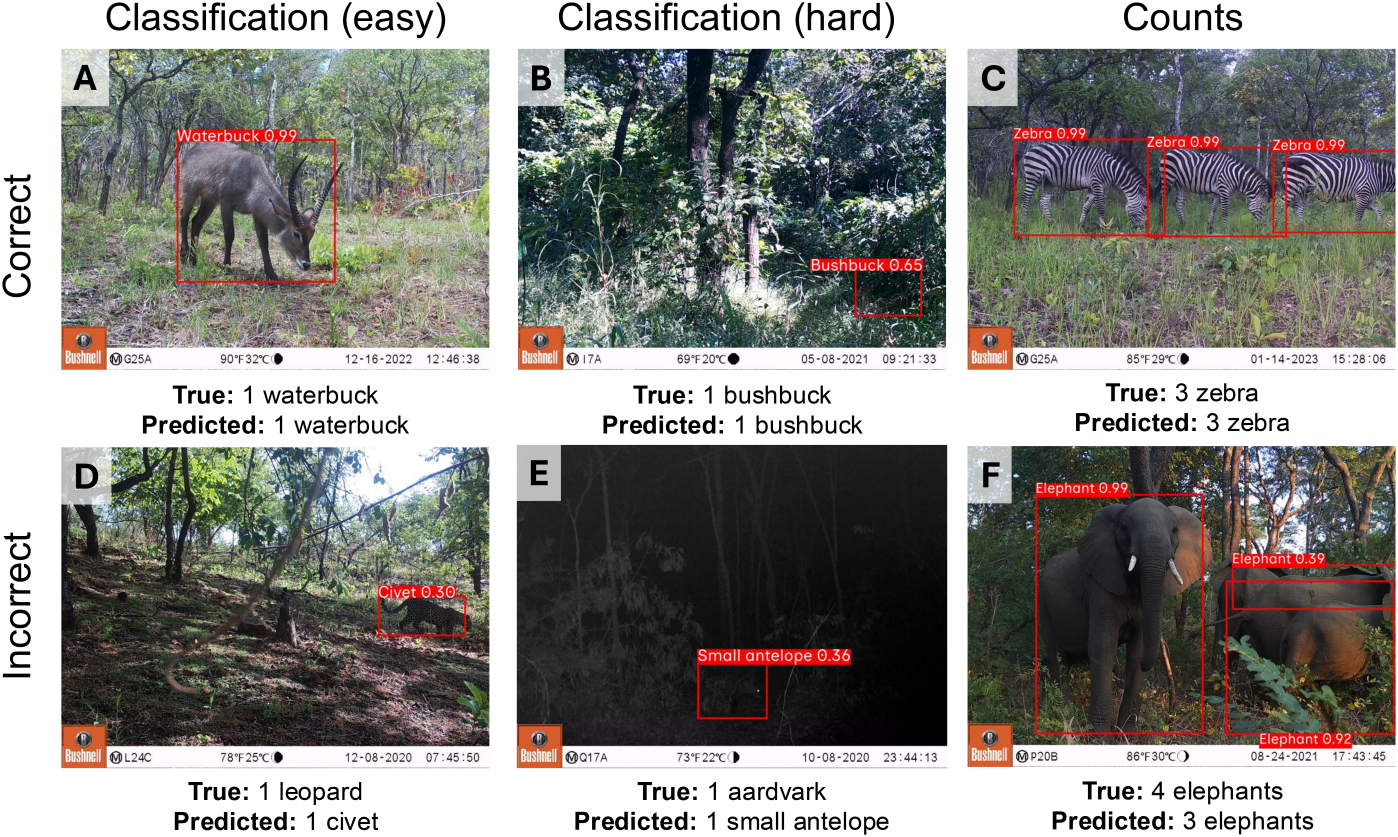
Examples of correct and incorrect predictions from a YOLOv4 multiclass detector model applied to camera trap images from Nkhotakota Wildlife Reserve, Malawi, from this study. (A) A clear example of a waterbuck, (B) a camouflaged bushbuck, (C) a group of three zebras, (D) a clear image of a leopard, (E) a nighttime image of an aardvark, and (F) a group of four elephants. Image credits: African Parks Network and USDA Forest Service.

We generated model predictions on approximately 3.5 million images from 158 cameras in 64 hexagons (mean = 22,204 images per camera, range = 89–53,956) (Figure 3). These images were collected after September 2020 and were not seen by the model during training. Of these, 299,385 images were predicted to contain animals (8.6%) and approximately 3.2 million were predicted to be empty. We reviewed 282,557 images as the independent test set for model evaluation, including all animal predictions with scores ≥0.25 (*n* = 119,733) and ≥100 predicted empty images from each camera deployment where possible (*n* = 162,824). The remaining approximately 3.2 million images not reviewed were also predicted to be empty or were excluded from evaluation; several camera deployments (*n* = 10) had between 2000 and 18,000 images remaining during review from a single class (baboon, bushbuck *Tragelaphus sylvaticus*, human, vervet monkey *Cercopithecus pygerythrus*, or warthog *Phacochoerus africanus*). Instead of reviewing all output in these instances, we reviewed ≥50 images from each of four confidence score bins (0–0.25, 0.26–0.50, 0.51–0.75, and 0.76–1.0) when possible and disregarded the rest from our evaluation. The test set also exhibited class imbalance (Figure 2).

#### Filtering empty images

**FIGURE 4:**
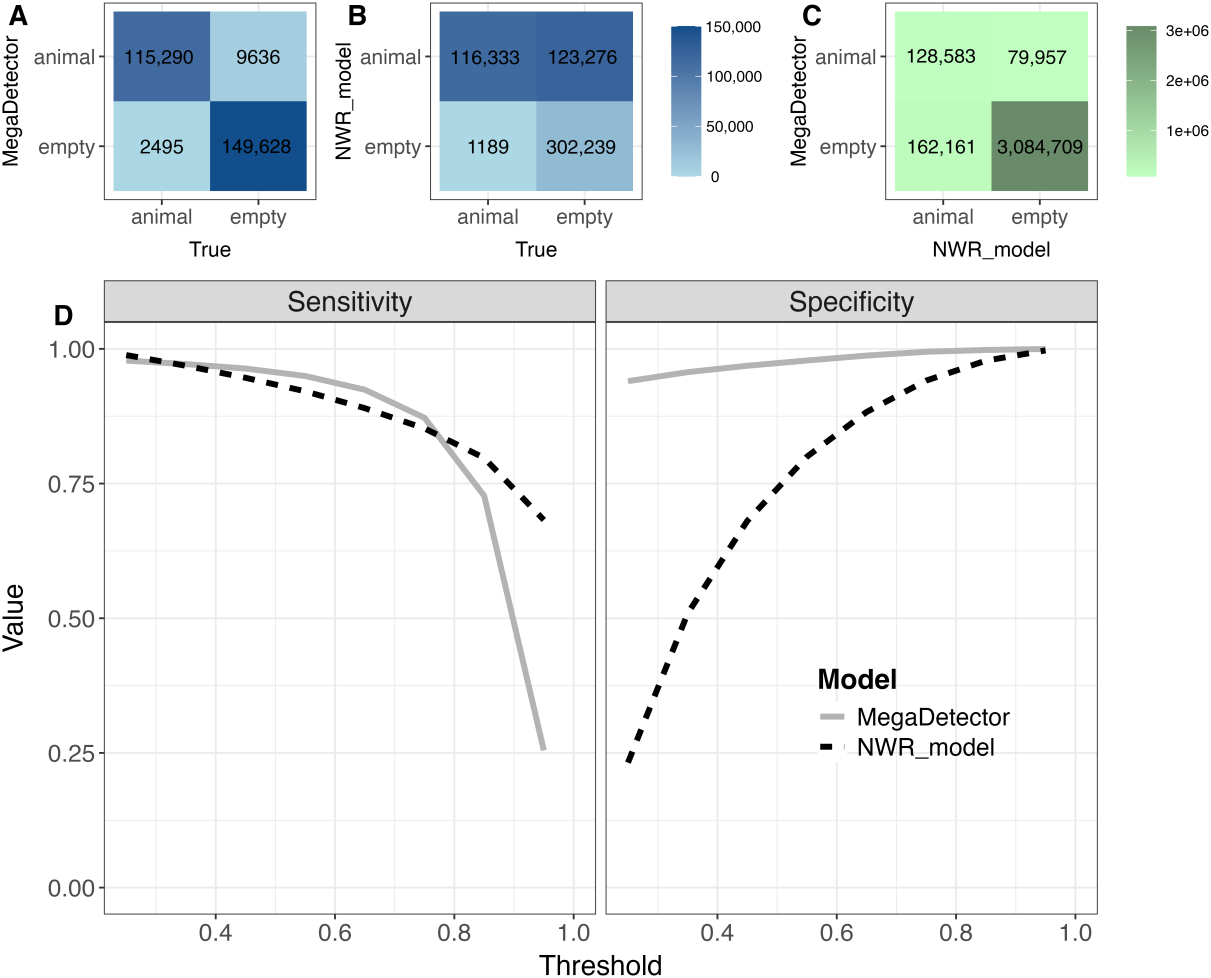
Confusion matrices comparing the predicted and actual number of images containing animals or empty according to (A) the MegaDetector v5 model (Beery et al., 2019) and (B) a YOLOv4 model trained on wildlife species from Nkhotakota Wildlife Reserve (NWR_model). Predictions of people, vehicles, and images with multiple classes are not included (these comprised <1% of the 282,557‐image test set). (C) Also shown are predictions by the MegaDetector and NWR models on the complete (not fully reviewed) dataset of approximately 3.5 million images. All detections were filtered using a score threshold of 0.25 for both models. (D) Performance by the two models at detecting animals at various score thresholds, where $\text{Sensitivity}=\text{True positives}/\left(\text{True positives}+\text{False positives}\right)$ and $\text{Specificity}=\text{True negatives}/\left(\text{True negatives}+\text{False positives}\right)$.

Our model had similar sensitivity to the MegaDetector v5 model but lower specificity (Figure 4D). Both models had sensitivity >0.75 except when the strictest thresholds were applied (e.g., scores >0.80), meaning that they performed well at finding images with animals in them. Our YOLO model had lower specificity than MegaDetector at all thresholds, suggesting that it incorrectly predicted animals in many images where there were none. For example, at a threshold of 0.25, our model‐predicted animals in 239,609 images, only 49% of which were correct, whereas of the 124,926 images with animal predictions by MegaDetector, 92% were correct (Figure 4A, B).

Because of its high sensitivity, the YOLO model missed fewer animals than MegaDetector (1189 compared to 2495 at a threshold of 0.25), but its lower specificity necessitated more manual review. We did not review images with scores <0.25 from our model, but at this threshold we reviewed approximately 1.4× as many images (*n* = 289,820) as we would have using MegaDetector predictions at the same threshold (*n* = 209,023), corresponding with 8.3% and 6.0% of the approximately 3.5 million processed images, respectively (Figure 4C).

The most commonly missed species were also the most prevalent species in our dataset (e.g., baboon and bushpig *Potamochoerus larvatus*) along with animals unidentifiable to species, but rarer species were missed at a higher proportion relative to their prevalence in the test dataset. Most FPs predicted by both models (i.e., objects falsely predicted to be animals) were tree branches, vegetation, or rocks.

#### Species classification

**FIGURE 5:**
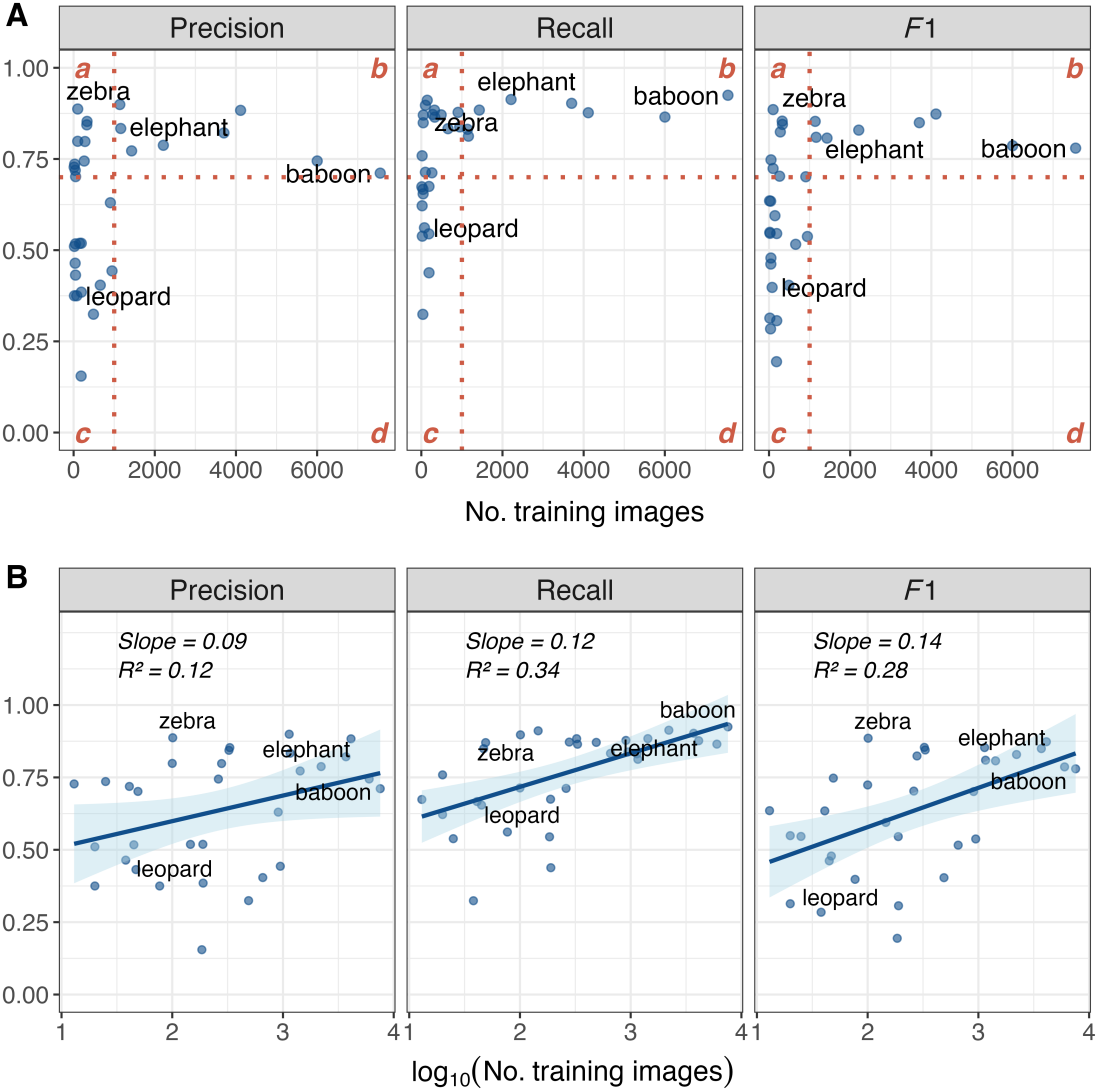
Performance metrics averaged for all confidence scores plotted against (A) the number of training images (B) and the log_10_ of training size for each class in a YOLOv4 multiclass detector model for wildlife species from Nkhotakota Wildlife Reserve, Malawi. Precision represents the proportion of model predictions that are correct detections (true positives), recall represents the proportion of true positives that are correctly predicted by the model, and *F*1 is a balanced metric where $F1=2\times \left(\text{Precision}\times \text{Recall}\right)/\left(\text{Precision}+\text{Recall}\right)$. As shown in panel (A), all classes with >1000 training images had precision, recall, and *F*1 scores >0.70 (quadrant *b*). Performance was more variable for classes with <1000 training images, with some performing well (those in quadrant *a*) and others performing relatively poorly (quadrant *c*). In panel (B), regression lines (estimates ± 95% CI) are from linear models with average precision, average recall, and average *F*1 as response variables and $\log_{10}(\text{Number of training images)}$ as the dependent variable.

Our model had overall macro‐averaged precision = 0.64, macro‐averaged recall = 0.76, and macro‐averaged *F*1 = 0.63, calculated for 32 species classes. We excluded classes with fewer than 10 images in the training or test sets from these calculations. Class‐specific performance was high for the eight classes that had >1000 training examples, with average precision, average recall, and average *F*1 values >0.70 (Figure 5; Appendix S1). Performance was more variable for classes with <1000 training examples: some classes had high performance despite few training examples (e.g., zebra *Equus burchelli*) while others had lower performance, as expected (e.g., leopard *Panthera pardus*) (Figure 5). Recall was highest for species with the most training examples, while precision (and therefore *F*1) was maximized at intermediate training data sizes (Figure 5). Overall, we found more support for a logarithmic relationship between the performance metrics and the number of training images per species than for a linear relationship (Figure 5; Appendix S2).

**FIGURE 6:**
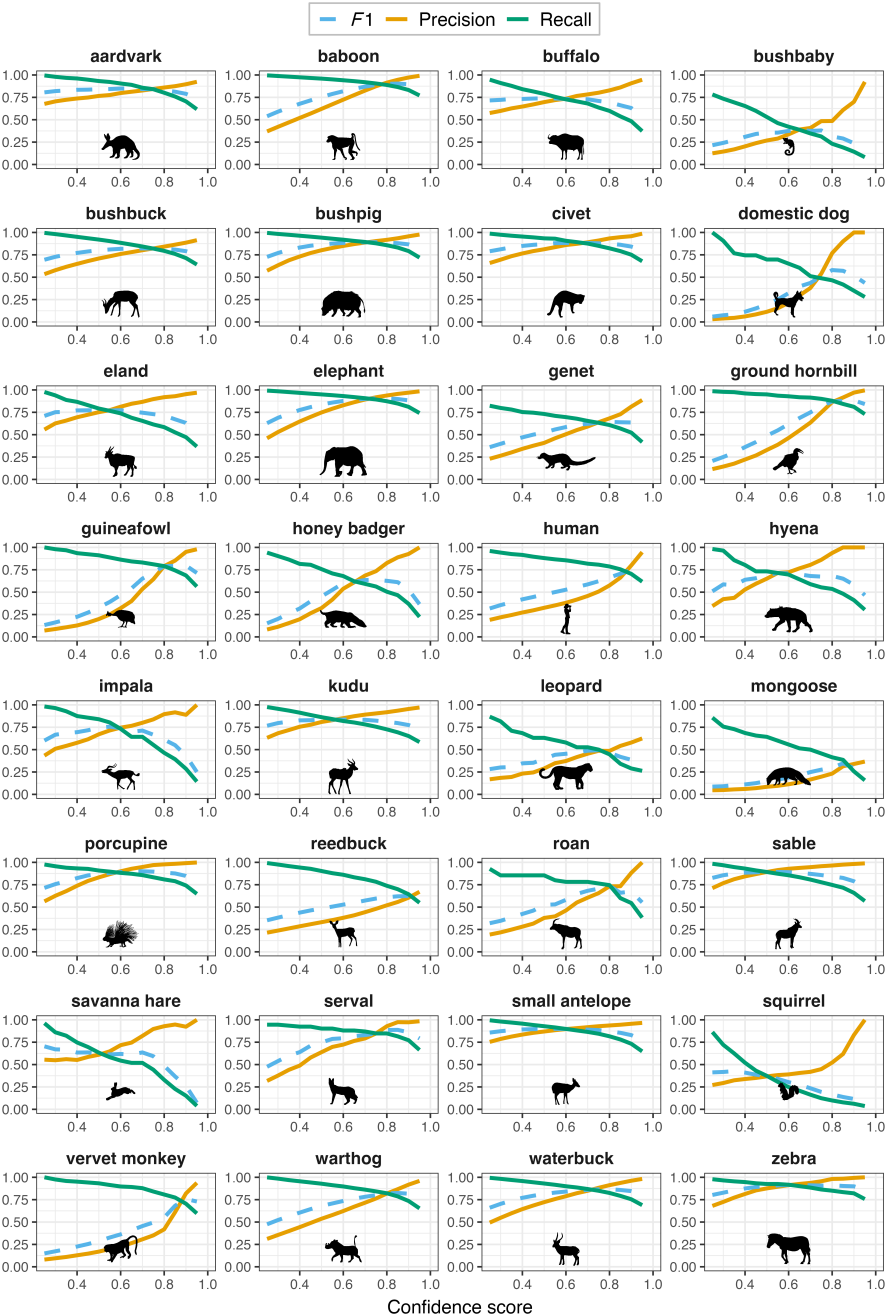
Performance metrics across confidence score thresholds for 32 classes in a YOLOv4 multiclass detector model for wildlife species from Nkhotakota Wildlife Reserve, Malawi. Image‐level precision (orange), recall (green), and *F*1 (blue dashed) are shown for classes with ≥10 examples in the training and evaluation datasets. Animal silhouettes are from PhyloPic (https://www.phylopic.org; T. Michael Keesey, 2025) and were added using the *rphylopic* R package (Gearty & Jones, 2023). Full attribution details are available at https://www.phylopic.org/permalinks/5b46138d9c0016fbef52e4d38eb5bcc84924caf1b31afc44ad179f77916bcf10.

Across species classes, recall was near 1.0 at the lowest confidence score threshold (0.25) but declined with increasingly strict score thresholds, as expected (Figure 6). Species classes with high recall (>0.80) at thresholds >0.90 included baboon, elephant, ground hornbill *Bucorvus leadbeateri*, and zebra. Conversely, precision increased with confidence score, indicating that the model assigned higher scores to TPs than FP images, which were filtered out at higher thresholds. All classes achieved precision >0.80 at thresholds >0.90 except leopard, mongoose spp., and reedbuck *Redunca arundinum* (Figure 6). Examining *F*1 scores suggests that the thresholds where performance was most balanced between precision and recall were highly variable among species. For example, *F*1 was maximized at thresholds >0.8 for some species (vervet monkey, baboon, serval *Leptailurus serval*, zebra) but peaked at approximately 0.6 or lower for others (buffalo *Syncerus caffer*, eland *Tragelaphus oryx*, impala *Aepyceros melampus*) (Figure 6).

For species with high average recall but low average precision, the model assigned high scores to both TPs and FPs (e.g., ground hornbill, guineafowl *Numida meleagris*, reedbuck, roan antelope *Hippotragus equinus*, vervet monkey) (Appendix S1). For species with high precision but low recall (e.g., eland, impala), the model assigned low scores to both TPs and FPs. Species with the best overall performance (e.g., zebra) were assigned high scores for TPs and low scores for FPs.

#### Species richness

**FIGURE 7:**
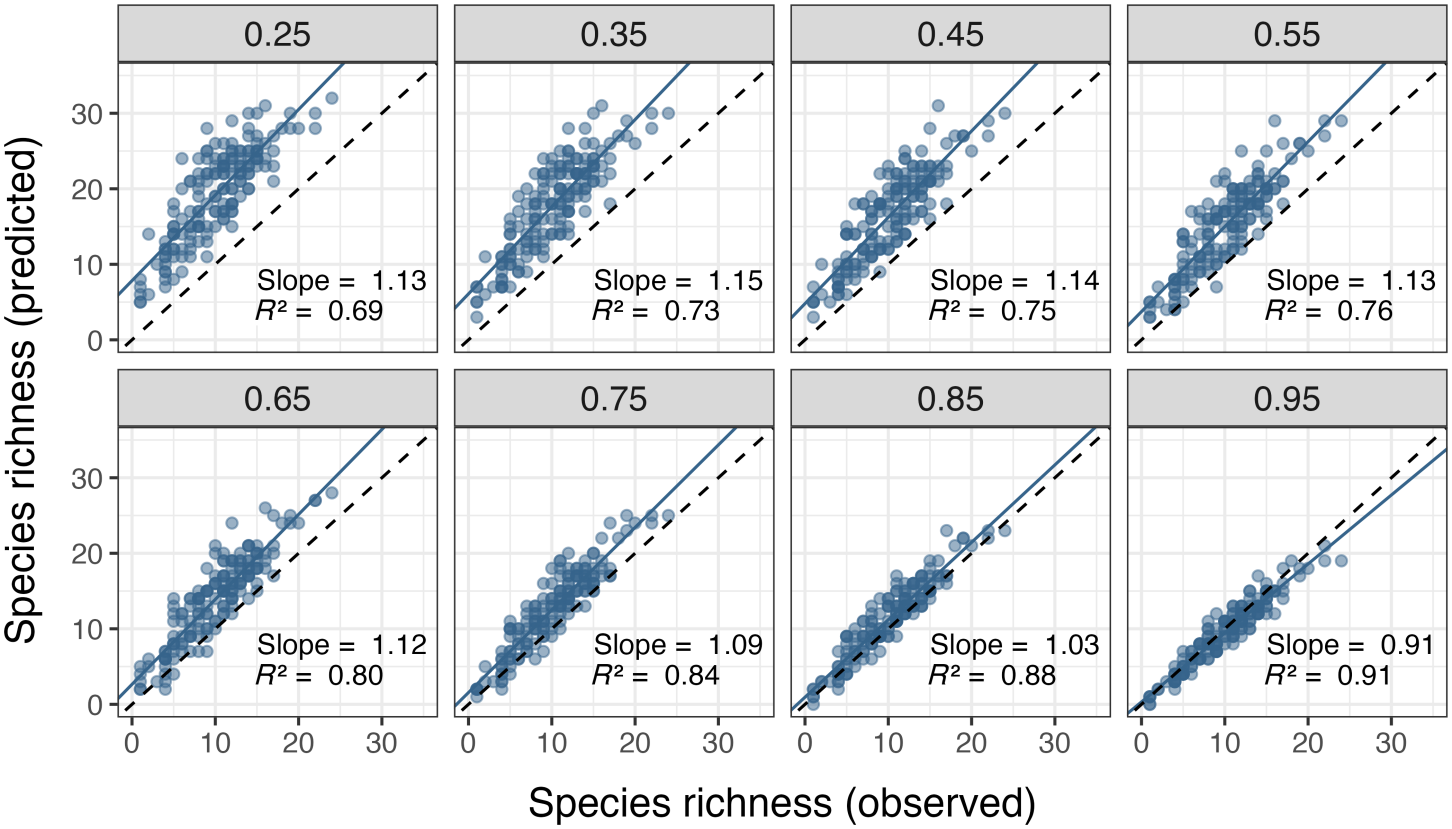
Comparison of species richness (number of species detected at each camera site, *n* = 158) calculated using model predictions at various confidence score thresholds (0.25–0.95) and species richness calculated using manually verified detections. The dashed line represents a 1:1 relationship. Slope and $R^{2}$ values are from a linear model with predicted richness as a function of observed richness.

Species richness was highly correlated between observed values using manually verified detections and predicted values using model outputs ($R^{2}$ = 0.69–0.91) (Figure 7; Appendix S3). The number of species detected at each camera location varied between 0 and 24 (mean = 10) using verified detections and between 0 and 32 (mean = 14) using model predictions. When score thresholds ≤0.85 were used to filter detections, richness was generally overestimated by model predictions (slope >1). The strongest correlation was achieved with score threshold 0.95 ($R^{2}$ = 0.91) (Figure 7). At score thresholds ≥0.85, mean absolute bias (across all 158 sites in the test set) was indistinguishable from zero based on SDs (threshold = 0.85: mean 1.20, SD 1.73; threshold = 0.95: mean −0.56, SD 1.37). In contrast, mean absolute bias for species richness was as high as 9.19 (SD 3.50) at the lowest score threshold of 0.25.

#### Animal counts

**FIGURE 8:**
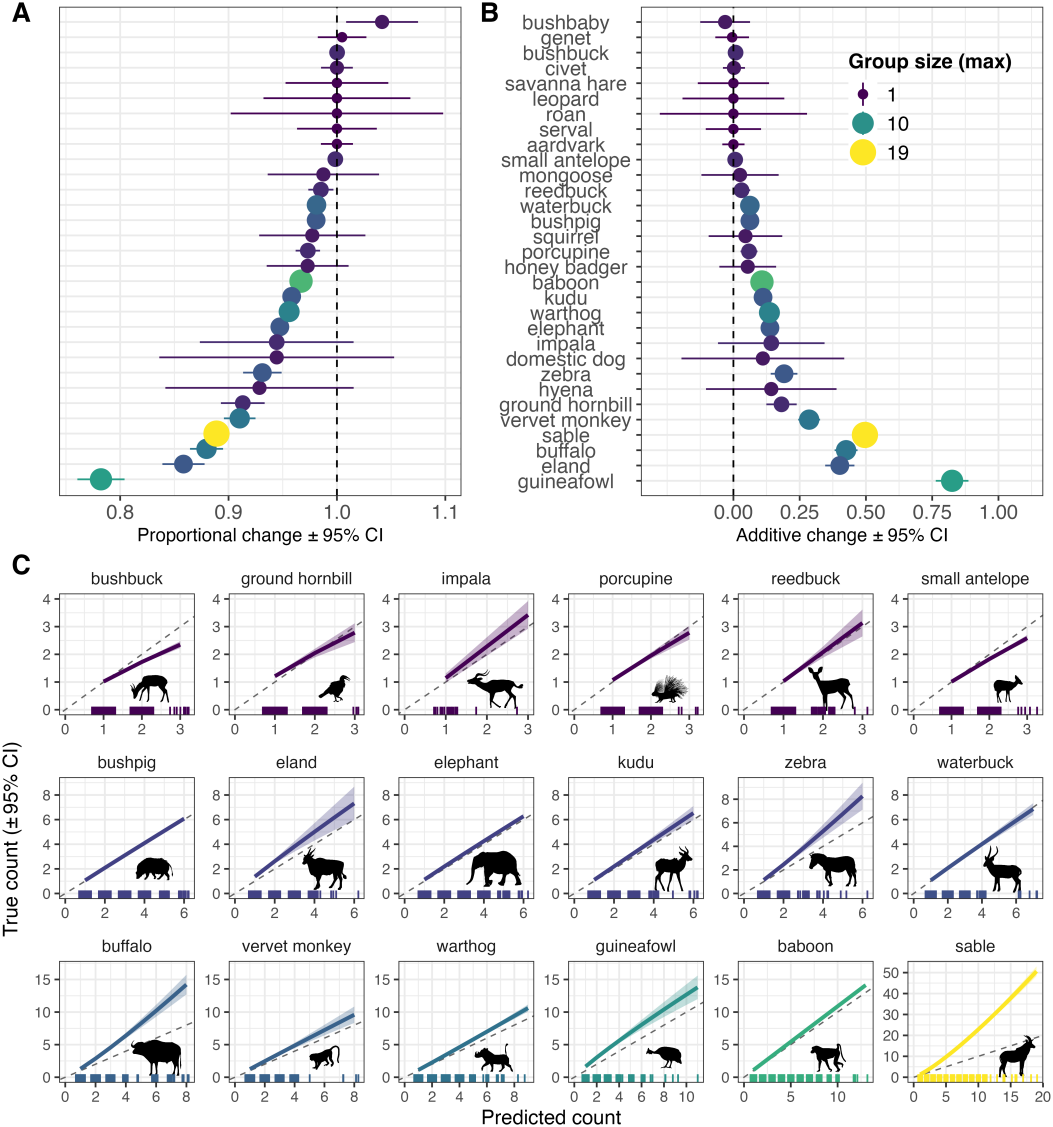
Estimates of the (A) proportional and (B) additive differences between true and predicted counts of animals in camera trap images using a YOLOv4 multiclass detector model for wildlife species in Nkhotakota Wildlife Reserve, Malawi. Points are coefficient estimates (±95% CI) from linear models with species class as the dependent variable and (A) ${\text{Count}}_{\text{predicted}}/{\text{Count}}_{\text{true}}$ and (B) ${\text{Count}}_{\text{true}}-{\text{Count}}_{\text{predicted}}$ as the response variables. Points are scaled and colored by the maximum observed group size per species class. (C) Also shown are the estimated relationships between true counts and model‐predicted counts for species with observed group sizes >2, where values above the 1:1 dashed line indicate that true counts are estimated to be higher than model‐predicted counts. Species are ordered and colored by increasing maximum observed group size. Note the different axis limits, which are based on observed group size ranges per species. Animal silhouettes are from PhyloPic (https://www.phylopic.org; T. Michael Keesey, 2025) and were added using the *rphylopic* R package (Gearty & Jones, 2023). Full attribution details are available at https://www.phylopic.org/permalinks/5b46138d9c0016fbef52e4d38eb5bcc84924caf1b31afc44ad179f77916bcf10.

Our model accurately counted the number of animals in images for approximately half of the species classes, but predicted counts were significantly lower than true counts for 15 of 32 species (*p* < 0.05) (Figure 8; Appendix S4). The greatest proportional differences were for species with the largest observed group sizes: guineafowl (mean group size 2.3, range 1–11), eland (mean 1.5, range 1–6), buffalo (mean 1.6, range 1–8), and sable antelope *Hippotragus niger* (mean 1.7, range 1–19), with underestimates of 22%, 14%, 12%, and 11%, respectively (Figure 8A). When slope was allowed to vary by species class, these differences were even more pronounced; for example, true and predicted counts were expected to vary by <1 animal for all species (Figure 8B), but they varied by as much as five animals using the model with ${\text{Count}}_{\text{predicted}}\times \text{Class}$ (Figure 8C). Animals missed by our model were often obscured or far from the cameras, and many would likely not have triggered the camera on their own (Figure 3).

## DISCUSSION

We demonstrate a workflow for training and deploying a custom computer vision model to detect and classify animals in camera trap images from Nkhotakota Wildlife Reserve, Malawi. Using our new, no‐code platform Njobvu‐AI (Koning et al., 2023) allowed us to annotate a modest‐sized dataset of 33,664 images, train a YOLO multiclass detector model, classify over 3 million new images, and collaboratively review model predictions. By training a preliminary model and using it to process new data as the monitoring program progressed, we could simultaneously evaluate initial model performance, identify images that can now be used for retraining to improve the model, and confirm animal detections for further analyses and reporting, in keeping with the priorities of the monitoring program. Such an approach will be particularly useful for programs that want to utilize computer vision for data processing but which may not have access to existing labeled datasets.

Despite its much smaller training base, our model successfully found images containing animals at a similar rate as MegaDetector, a commonly used model designed to be generalizable at the object‐detection step (Beery et al., 2019). However, our model was not as specific at filtering images without animals, resulting in more effort to review FPs. Our training and test images are more similar in distribution to each other than to the millions of images used to train MegaDetector, contributing to high sensitivity, but our model's lower specificity with filtering false triggers is likely due to the relatively small proportion of empty images used for training. Although MegaDetector is able to achieve relatively high sensitivity and specificity, for small datasets, prioritizing sensitivity—or not falsely missing images of animals—is often a higher priority for practitioners than maximizing specificity. The high sensitivity to animals that we observed suggests that custom computer vision models can be used at the initial stages of a project as an effective first step for filtering empty images and then updated iteratively to improve specificity and species‐specific classification performance.

While MegaDetector is a valuable resource for camera trap users, other image modalities do not have existing domain‐specific models. In these cases, training custom detectors using the workflow presented here may be especially beneficial. Many camera trap users, however, may find little benefit relative to annotation effort in training custom detectors over using MegaDetector for the purpose of filtering empty images. The approach we present here goes a step further by predicting species classification simultaneously with bounding‐box detection. Another commonly used approach is training whole‐image classifiers (e.g., Gomez Villa et al., 2017; Norouzzadeh et al., 2021), which requires only image‐level labels rather than bounding boxes, which is more typical of existing labeled camera trap datasets. In fact, a two‐stage approach using a generalized detector to locate objects and then a custom classifier trained on the bounding‐box crops has been shown to offer advantages over either method (Gadot et al., 2024). This pipeline will be a critical feature to embed within future versions of Njobvu‐AI.

Nevertheless, the utility of preliminary multiclass detector models is illustrated by our model's species‐specific classification performance, which was high for species with the most training examples but otherwise varied considerably and depended on the confidence score threshold used. The variability in classification performance of our model followed some predictable trends. Our model achieved average precision, recall, and *F*1 scores >0.70 for all classes with >1000 training images but also for some species with as few as 49 and 101 images (serval and zebra, respectively). Notably, these are species with distinct morphological features that had clear examples in the training dataset, which contributed to high performance despite few examples. While recall is expected to increase with more training examples as the model becomes better at locating TPs, precision is not necessarily monotonically related to training data size because models can become overfit to common classes, thus producing more FPs. This explains our findings that precision was maximized for species with intermediate training data sizes and that the relationship between performance metrics and training data size most closely followed a logarithmic rather than linear trend. Other studies using camera trap data have also found either a logarithmic relationship between precision or recall and the number of training images (Schneider et al., 2020; Shahinfar et al., 2020; Tabak et al., 2019) or no relationship (Duggan et al., 2021), but optimal reported training data sizes are highly variable and project dependent. Regardless, although local models generally use data more closely aligned with the prevalence of species in the environment, detecting rare species continues to be a challenge for camera trap studies when few training examples are available, even for otherwise high‐performing models. Long‐tailed distributions are common in ecological datasets, and developing models to effectively predict on datasets with high class imbalance is an ongoing topic in deep learning research (Buda et al., 2017; Cunha et al., 2023; Yang et al., 2021).

We also observed trends in classification performance related to confidence scores, with precision generally increasing and recall decreasing with progressively stricter score thresholds. Our threshold‐specific performance metrics can be used to inform review procedures based on acceptable risks of missing TPs balanced with the additional effort to review FPs (Villon et al., 2020). These decisions will likely be made on a class‐level basis, depending on the importance of recall or precision for each species. For example, using a threshold of 0.85 for review of baboon images would capture most TPs while requiring few false positives to be reviewed; in contrast, a much more conservative threshold would be needed to ensure high recall for rarer species like impala and leopard.

In terms of ecologically relevant metrics, we found acceptable levels of agreement between verified and model‐predicted species richness and per‐image animal counts. Our species richness estimates from raw model predictions were correlated with estimates using confirmed detections, especially at high score thresholds. Unlike findings by Whytock et al. (2021), our model had a tendency to overestimate the number of species except at high confidence thresholds, at which bias approached zero. Depending on the importance of accurate species richness measurements, these estimates may be a useful basis for further analysis with even minimal manual review. Similarly, our model accurately predicted the number of animals in each image for approximately half of the species classes, especially those occurring singly or in pairs. For group‐living species, the model generally underestimated the number of animals in images (e.g., missing some buffalo out of a herd) but these counts differed by 22% or less. For analyses requiring group size data (e.g., random encounter models to estimate animal density; Rowcliffe et al., 2008), counts may need to be manually verified or corrected using model coefficients for the estimated relationships between model predictions and true counts. Counting animals in images has been an area of interest in camera trap studies (Norouzzadeh et al., 2017; Tabak et al., 2019) as well as for aerial imagery—for example, to survey mammal herds or bird colonies (Delplanque et al., 2023; Hodgson et al., 2018; Kellenberger et al., 2019). In all modalities, accurate counting is difficult due to overlap, occlusion, and lower probability of detecting animals far from the sensors. Additionally, animal groups are often not entirely captured within single images, and this varies by animal body size and movement speed. Counting may be more appropriately attempted by considering sequences or videos rather than individual images, or future computer vision models may treat this as an object tracking task.

Studies using off‐the‐shelf computer vision models to detect and classify animals often observe poor performance due to the limited generalizability of models trained in one system and applied to another. Custom models generally have higher performance due to greater similarity in distribution between images in the training and test data; however, considering expectations of generalizability is also important when developing and evaluating custom models. Our goal was to train a model generalizable to future years of the wildlife monitoring program at Nkhotakota Wildlife Reserve, including camera locations not seen during training. Best practices in this situation generally dictate careful splitting to ensure model performance is optimized for the desired use case—ensuring similar distributions in training and test data among species, locations, time of day, and seasons, which are important sources of visual variation (Beery et al., 2018). On the other hand, researchers faced with relatively small ecological datasets are often not able to achieve realistic splits, instead prioritizing using all available data in model training. Using a relatively naïve splitting approach, we achieved temporal independence (no images from the same deployments were in both training and test data) as well as partial spatial independence, but there were some substantial differences in class distribution among splits. Our model's performance could likely be improved by more intentional data curation with regard to species classes and visual variation. While our model may also generalize somewhat to datasets with similar species composition and miombo woodland environment, our approach allowed us to develop a model optimized for the monitoring program in Nkhotakota Wildlife Reserve and evaluate its performance relative to project‐specific priorities and objectives.

Implementing computer vision models has become easier with the increased availability of user‐friendly tools and platforms, including many for camera trap data (Tuia et al., 2022; Vélez et al., 2023). However, options to train custom models are still primarily limited to programming packages rather than user‐friendly interfaces (but see Kellenberger et al., 2020 and Project Zamba https://zamba.drivendata.org). Using Njobvu‐AI allowed us to complete all steps of this process in one no‐code platform, from labeling images, training the model, classifying new data, and reviewing model predictions. This tool provides many features that users rely on in existing labeling tools, including features for collaboration and custom control of data, while offering a gentle introduction to model training. To promote responsible use of computer vision models, tools like this are best used in combination with dedicated training in machine learning concepts. Njobvu‐AI is an open‐source tool in active development under guidance from multiple ecological research groups (https://github.com/sullichrosu/Njobvu-AI). The development version supports newer model architectures than what we used here (YOLO11), and future releases should prioritize the implementation of additional features including more data curation and input options and integration with existing models like MegaDetector. We envision this tool to be a valuable addition to research and monitoring programs by increasing the efficiency of data processing and reducing the time between collection and analysis.

## AUTHOR CONTRIBUTIONS

**Cara L. Appel:** Conceptualization; methodology; software; validation; formal analysis; investigation; data curation; writing—original draft; and visualization. **Ashwin Subramanian:** Methodology and software. **Jonathan S. Koning:** Methodology and software. **Marnet Ngosi:** Methodology; investigation; data curation; project administration. **Christopher M. Sullivan:** Conceptualization; methodology; software; resources; supervision, and project administration. **Taal Levi:** Conceptualization; methodology; investigation; resources; writing—review and editing; supervision; project administration; and funding acquisition. **Damon B. Lesmeister:** Conceptualization; methodology; investigation; resources; writing—review and editing; supervision; project administration; and funding acquisition.

## CONFLICT OF INTEREST STATEMENT

The authors declare no conflicts of interest.

## Supporting information


Appendix S1.

Appendix S2.

Appendix S3.

Appendix S4.

## Data Availability

Source code and documentation for Njobvu‐AI (Appel et al., 2024) are available in Zenodo at https://doi.org/10.5281/zenodo.14167280. Data and code to reproduce the model evaluation (Appel, 2025) are available in Zenodo at https://doi.org/10.5281/zenodo.15997860. Images used for model training and evaluation are archived in LILA BC at https://lila.science/datasets/nkhotakota-camera-traps.
