# Supplementary material for: Developing custom computer vision models with Njobvu‐AI: A collaborative, user‐friendly platform for ecological research
Source: Ecol Appl. 2025 Sep 11;35(6):e70096. doi: 10.1002/eap.70096 (PMC12426366; doi:10.1002/eap.70096)
Supplement: Supplementary file 1 — Appendix S1. [file EAP-35-e70096-s002.pdf]

APPENDIX S1. Distribution of model classes by data split

Title: Developing custom computer vision models with Njobvu-AI: A collaborative, user-friendly platform for ecological research

Authors: Cara L. Appel, Ashwin Subramanian, Jonathan S. Koning, Marnet Ngosi, Christopher M. Sullivan, Taal Levi, Damon B. Lesmeister

Journal: Ecological Applications

Table S1. Wildlife taxa in camera trap images from Nkhotakota Wildlife Reserve, Malawi. The first 37 animal classes were used to train a YOLOv4 multiclass detector model and are shown with their average precision, average recall, and average F1 scores across confidence thresholds from 0.25 to 0.95 (incremented by 0.05). Four additional classes were identified during review of images but were not included in model training.

Class name	Taxonomic description	Images in training set	Images in test set	Average precision	Average recall	Average F1
Model classes						
Aardvark	*Orycteropus afer*	279	601	0.80	0.87	0.82
Baboon	Yellow Baboon *Papio cynocephalu*s	7555	44198	0.71	0.92	0.78
Bat	Chiroptera spp.	1	2	NA*	NA*	NA*
Blue Monkey	*Cercopithecus mitis*	4	0	NA*	NA*	NA*
Buffalo^	Cape Buffalo *Syncerus caffer*	261	695	0.74	0.71	0.70
Bushbaby	Galago or Bushbaby (Galagidae spp.)	190	151	0.38	0.44	0.31

Bushbuck	Cape Bushbuck *Tragelaphus sylvaticus*	6002	10488	0.74	0.86	0.79
Bushpig	*Potamochoerus larvatus*	3704	10713	0.82	0.90	0.85
Cattle	Domestic cattle	24	0	NA*	NA*	NA*
Civet	African Civet *Civettictis civetta*	325	554	0.84	0.88	0.85
Dog	Domestic dog	32	37	0.37	0.62	0.31
Eland^	Common Eland *Taurotragus oryx*	100	472	0.80	0.71	0.72
Elephant^	African Savanna Elephant *Loxodonta africana*	2211	7643	0.79	0.91	0.83
Empty		968	162,824	NA	NA	NA
Genet	Genetta sp.	189	293	0.52	0.67	0.55
Goat	Domestic goat	3	0	NA*	NA*	NA*
Ground Hornbill	Southern Ground Hornbill *Bucorvus leadbeateri*	146	289	0.52	0.91	0.59
Guineafowl	Helmeted Guineafowl *Numida meleagris*	47	258	0.43	0.85	0.48
Honey Badger	*Mellivora capensis*	45	104	0.52	0.65	0.46
Human		946	1739	0.44	0.84	0.54
Hyena	Spotted Hyena *Crocuta crocuta*	41	61	0.72	0.67	0.63
Impala^	*Aepyceros melampus*	13	91	0.73	0.67	0.64
Jackal	Side-Striped Jackal *Lupulella adustus*	9	56	NA*	NA*	NA*
Kudu^	Greater Kudu *Tragelaphus strepsiceros*	1161	2661	0.83	0.81	0.81
Leopard	*Panthera pardus*	77	39	0.37	0.56	0.40
Mongoose	Herpestidae spp.	185	74	0.15	0.54	0.19

Porcupine	Cape Porcupine *Hystrix africaeaustralis*	331	933	0.85	0.86	0.84
Reedbuck	Southern Reedbuck *Redunca arundinum*	657	1066	0.40	0.83	0.52
Roan Antelope	*Hippotragus equinus*	20	72	0.51	0.76	0.55
Sable Antelope^	*Hippotragus niger*	1138	5184	0.90	0.83	0.85
Savanna Hare	*Lepus victoriae*	25	80	0.74	0.54	0.55
Serval	*Leptailurus serval*	49	93	0.70	0.87	0.75
Small antelope	Common Duiker *Sylvicapra grimmia*, Klipspringer *Oreotragus oreotragus*, Red Duiker *Cephalophus harveyi*, Sharpe's Grysbok *Raphicerus sharpei*	4114	21142	0.88	0.88	0.87
Squirrel	Sciuridae spp.	38	756	0.46	0.32	0.28
Vervet Monkey	*Cercopithecus pygerythrus*	489	566	0.32	0.87	0.40
Warthog^	Common Warthog *Phacochoerus africanus*	906	3973	0.63	0.88	0.70
Waterbuck^	Common Waterbuck *Kobus ellipsiprymnus*	1429	3363	0.77	0.88	0.81
Zebra^	Plains Zebra *Equus burchelli*	101	384	0.89	0.90	0.89
Additional classes						
Other animal	Unidentifiable animals or taxa not listed elsewhere	NA	867	--	--	--
Other bird	Bird taxa not listed elsewhere	NA	220	--	--	--
Palm civet	African Palm Civet *Nandinia binotata*	NA	34	--	--	--
Small mammal	e.g., Rodents	NA	26	--	--	--

^ Indicates species that were part of the translocation effort.

* Species classes with < 10 images in either training or test data were excluded from macro-averaged precision, macro-averaged recall, and macro-averaged F1 score calculations.

Table S2. Distribution of species classes in training, validation, and test splits for a multiclass detector model to identify wildlife species in camera trap images from Nkhotakota Wildlife Reserve, Malawi.

Class	Split	Number of images	Number of unique sites	Proportion of split
aardvark	training	279	26	0.008
aardvark	validation	26	8	0.003
aardvark	test	601	52	0.005
baboon	training	7555	37	0.230
baboon	validation	2366	37	0.292
baboon	test	44198	148	0.372
bat_sp.	training	1	1	< 0.001
bat_sp.	validation	0	0	NA
bat_sp.	test	2	2	< 0.001
blue_monkey	training	4	1	< 0.001
blue_monkey	validation	4	2	< 0.001
blue_monkey	test	0	0	NA
buffalo	training	261	17	0.008
buffalo	validation	124	6	0.015
buffalo	test	695	34	0.006
bush_squirrel	training	38	2	0.001
bush_squirrel	validation	0	0	NA
bush_squirrel	test	756	1	0.006
bushbaby	training	190	17	0.006
bushbaby	validation	5	3	0.001
bushbaby	test	151	28	0.001
bushbuck	training	6002	78	0.183
bushbuck	validation	572	18	0.070
bushbuck	test	10488	113	0.088
bushpig	training	3704	92	0.113
bushpig	validation	316	31	0.039

bushpig	test	10713	134	0.090
civet	training	325	47	0.010
civet	validation	35	11	0.004
civet	test	554	63	0.005
domestic_cattle	training	24	1	0.001
domestic_cattle	validation	0	0	NA
domestic_cattle	test	0	0	NA
domestic_dog	training	32	7	0.001
domestic_dog	validation	2	2	< 0.001
domestic_dog	test	37	12	< 0.001
eland	training	100	11	0.003
eland	validation	6	2	0.001
eland	test	472	30	0.004
elephant	training	2211	57	0.067
elephant	validation	812	28	0.100
elephant	test	7643	87	0.064
genet	training	189	27	0.006
genet	validation	19	5	0.002
genet	test	293	32	0.002
goat	training	3	1	< 0.001
goat	validation	0	0	NA
goat	test	0	0	NA
ground_hornbill	training	146	28	0.004
ground_hornbill	validation	35	13	0.004
ground_hornbill	test	289	38	0.002
guinea_fowl	training	47	11	0.001
guinea_fowl	validation	3	3	0.000
guinea_fowl	test	258	17	0.002
honey_badger	training	45	15	0.001
honey_badger	validation	4	3	< 0.001
honey_badger	test	104	25	0.001
human	training	946	101	0.029
human	validation	383	44	0.047
human	test	1739	136	0.015
hyena	training	41	9	0.001
hyena	validation	12	1	0.001
hyena	test	61	10	0.001
impala	training	13	3	< 0.001
impala	validation	3	2	< 0.001
impala	test	91	9	0.001

kudu	training	1161	32	0.035
kudu	validation	440	25	0.054
kudu	test	2661	53	0.022
leopard	training	77	8	0.002
leopard	validation	9	1	0.001
leopard	test	39	12	< 0.001
mongoose	training	185	18	0.006
mongoose	validation	6	1	0.001
mongoose	test	74	18	0.001
porcupine	training	331	37	0.010
porcupine	validation	67	15	0.008
porcupine	test	933	62	0.008
reedbuck	training	657	44	0.020
reedbuck	validation	284	19	0.035
reedbuck	test	1066	46	0.009
roan	training	20	7	0.001
roan	validation	3	2	0.000
roan	test	72	14	0.001
sable	training	1138	45	0.035
sable	validation	441	19	0.054
sable	test	5184	81	0.044
scrub_hare	training	25	7	0.001
scrub_hare	validation	0	0	NA
scrub_hare	test	80	12	0.001
servalidation	training	49	11	0.001
servalidation	validation	0	0	NA
servalidation	test	93	22	0.001
side-striped_jackal	training	9	5	< 0.001
side-striped_jackal	validation	0	0	NA
side-striped_jackal	test	56	9	< 0.001
small_antelope	training	4114	106	0.125
small_antelope	validation	1303	39	0.161
small_antelope	test	21142	150	0.178
vervet_monkey	training	489	11	0.015
vervet_monkey	validation	23	2	0.003
vervet_monkey	test	566	12	0.005
warthog	training	906	61	0.028
warthog	validation	192	21	0.024
warthog	test	3973	90	0.033
waterbuck	training	1429	40	0.044

waterbuck	validation	302	19	0.037
waterbuck	test	3363	51	0.028
zebra	training	101	8	0.003
zebra	validation	4	4	< 0.001
zebra	test	384	19	0.003
