# Supplementary material for: Developing custom computer vision models with Njobvu‐AI: A collaborative, user‐friendly platform for ecological research
Source: Ecol Appl. 2025 Sep 11;35(6):e70096. doi: 10.1002/eap.70096 (PMC12426366; doi:10.1002/eap.70096)
Supplement: Supplementary file 2 — Appendix S2. [file EAP-35-e70096-s001.pdf]

APPENDIX S2. Model results for classification performance

Title: Developing custom computer vision models with Njobvu-AI: A collaborative, user-friendly platform for ecological research

Authors: Cara L. Appel, Ashwin Subramanian, Jonathan S. Koning, Marnet Ngosi, Christopher M. Sullivan, Taal Levi, Damon B. Lesmeister

Journal: Ecological Applications

Results summaries from linear models to assess the relationship between classification performance (average precision, average recall, and average F1 across confidence score thresholds) and the number of training images per class in a YOLOv4 multiclass detector for wildlife species in Nkhotakota Wildlife Reserve, Malawi. Models were run using the *lm* function in Program R.

LINEAR RELATIONSHIP MODELS

Average Precision (AP):

Call: lm(formula = AP ~ train_images, data = avg_metrics_filtered)

Residuals: Min (-0.4508), 1Q (-0.1553), Median (0.0464), 3Q (0.1373), Max (0.2844)
Residual standard error: 0.1944 on 30 degrees of freedom
Multiple R-squared: 0.09867
Adjusted R-squared: 0.06862
F-statistic: 3.284 on 1 and 30 DF, p-value: 0.07998

	Estimate	Std. Error	t value	Pr(>|t|)
(Intercept)	5.992e-01	3.959e-02	15.135	1.38e-15
train_images	3.477e-05	1.919e-05	1.812	0.08

Average Recall (AR):

Call: lm(formula = AR ~ train_images, data = avg_metrics_filtered)

Residuals: Min (-0.4053), 1Q (-0.0726), Median (0.0365), 3Q (0.11814), Max (0.1779)
Residual standard error: 0.143 on 30 degrees of freedom
Multiple R-squared: 0.1744,
Adjusted R-squared: 0.1469
F-statistic: 6.338 on 1 and 30 DF, p-value: 0.01739

	Estimate	Std. Error	t value	Pr(>|t|)
(Intercept)	7.280e-01	2.913e-02	24.993	<2e-16
train_images	3.554e-05	1.412e-05	2.517	0.0174

Average F1 (AF1):

Call: lm(formula = AF1 ~ train_images, data = avg_metrics_filtered)

Residuals: Min (-0.4003), 1Q (-0.1138), Median (0.0242), 3Q (0.14428), Max (0.2948)
Residual standard error: 0.1855 on 30 degrees of freedom
Multiple R-squared: 0.176,
Adjusted R-squared: 0.1485
F-statistic: 6.406 on 1 and 30 DF, p-value: 0.01685

	Estimate	Std. Error	t value	Pr(>|t|)
(Intercept)	5.859e-01	3.778e-02	15.507	7.22e-16
train_images	4.635e-05	1.831e-05	2.531	0.0169

LOGARITHMIC RELATIONSHIP MODELS

Average Precision (AP):

Call: lm(formula = AP ~ log10(train_images), data = avg_metrics_filtered)

Residuals: Min (-0.4677), 1Q (-0.1129), Median (0.0290), 3Q (0.1555), Max (0.2878)
Residual standard error: 0.1924 on 30 degrees of freedom
Multiple R-squared: 0.1171,
Adjusted R-squared: 0.08764

F-statistic: 3.978 on 1 and 30 DF, p-value: 0.05526

	Estimate	Std. Error	t value	Pr(>|t|)
(Intercept)	0.42204	0.11196	3.770	0.000716
log10(train_images)	0.08842	0.04433	1.994	0.055258

Average Recall (AR):

Call: lm(formula = AR ~ log10(train_images), data = avg_metrics_filtered)

Residuals: Min (-0.3446), 1Q (-0.0346), Median (-0.0002), 3Q (0.0771), Max (0.1888)
Residual standard error: 0.1279 on 30 degrees of freedom
Multiple R-squared: 0.3401
Adjusted R-squared: 0.3181
F-statistic: 15.46 on 1 and 30 DF, p-value: 0.0004604

	Estimate	Std. Error	t value	Pr(>|t|)
(Intercept)	0.48565	0.07441	6.527	3.23e-07
log10(train_images)	0.11585	0.02946	3.932	0.00046

Average F1 (AF1):

Call: lm(formula = AF1 ~ log10(train_images), data = avg_metrics_filtered)

Residuals: Min (-0.4204), 1Q (-0.0940), Median (0.0539), 3Q (0.1142), Max (0.3064)
Residual standard error: 0.1739 on 30 degrees of freedom
Multiple R-squared: 0.2763
Adjusted R-squared: 0.2521
F-statistic: 11.45 on 1 and 30 DF, p-value: 0.002006

	Estimate	Std. Error	t value	Pr(>|t|)
(Intercept)	0.30721	0.10118	3.036	0.00492
log10(train_images)	0.13559	0.04007	3.384	0.00201
