# Supplementary material for: Developing custom computer vision models with Njobvu‐AI: A collaborative, user‐friendly platform for ecological research
Source: Ecol Appl. 2025 Sep 11;35(6):e70096. doi: 10.1002/eap.70096 (PMC12426366; doi:10.1002/eap.70096)
Supplement: Supplementary file 3 — Appendix S3. [file EAP-35-e70096-s004.pdf]

APPENDIX S3. Model results for species richness

Title: Developing custom computer vision models with Njobvu-AI: A collaborative, user-friendly platform for ecological research

Authors: Cara L. Appel, Ashwin Subramanian, Jonathan S. Koning, Marnet Ngosi, Christopher M. Sullivan, Taal Levi, Damon B. Lesmeister

Journal: Ecological Applications

Results summaries from linear models to compare species richness using confirmed detections (rich_obs) and model predictions (rich_pred) from a YOLOv4 multiclass detector for wildlife species in Nkhotakota Wildlife Reserve, Malawi. Models were run using the *lm* function in Program R. Each model used a different score threshold for filtering predicted detections.

SPECIES RICHNESS MODELS

Call: lm(formula = rich_pred ~ rich_obs, data = richness_thresh)

threshold = 0.25:

Residuals: Min (-6.9063), 1Q (2.5230), Median (-0.4841), 3Q (2.3661), Max (10.1834)
Residual standard error: 3.561 on 156 degrees of freedom
Multiple R-squared: 0.6904
Adjusted R-squared: 0.6884
F-statistic: 347.8 on 1 and 156 DF, p-value: < 2.2e-16

	Estimate	Std. Error	t value	Pr(>|t|)
(Intercept)	8.03830	0.68137	11.80	<2e-16
rich_obs	1.15566	0.06197	18.65	<2e-16

threshold = 0.35:

Residuals: Min (-8.1216), 1Q (-2.5976), Median (-0.3843), 3Q (2.2584), Max (9.8615)
Residual standard error: 3.285 on 156 degrees of freedom
Multiple R-squared: 0.7266
Adjusted R-squared: 0.7248
F-statistic: 414.5 on 1 and 156 DF, p-value: < 2.2e-16

	Estimate	Std. Error	t value	Pr(>|t|)
(Intercept)	6.33628	0.62854	10.08	<2e-16
rich_obs	1.16384	0.05716	20.36	<2e-16

threshold = 0.45:

Residuals: Min (-6.5281), 1Q (-2.3239), Median (-0.4333), 3Q (2.1707), Max (8.6178)
Residual standard error: 3.071 on 156 degrees of freedom
Multiple R-squared: 0.7467
Adjusted R-squared: 0.7451
F-statistic: 459.8 on 1 and 156 DF, p-value: < 2.2e-16

	Estimate	Std. Error	t value	Pr(>|t|)
(Intercept)	5.04661	0.58761	8.588	<2e-16
rich_obs	1.14597	0.05344	21.444	<2e-16

threshold = 0.55:

Residuals: Min (-5.9955), 1Q (-1.8739), Median (-0.4927), 3Q (1.9731), Max (7.9991)
Residual standard error: 2.877 on 156 degrees of freedom
Multiple R-squared: 0.7642
Adjusted R-squared: 0.7627
F-statistic: 505.5 on 1 and 156 DF, p-value: < 2.2e-16

	Estimate	Std. Error	t value	Pr(>|t|)
(Intercept)	3.99002	0.55050	7.248	<2e-16
rich_obs	1.12568	0.05007	22.484	<2e-16

threshold = 0.65:

Residuals: Min (-4.9364), 1Q (-1.6539), Median (-0.3534), 3Q (1.8506), Max (7.6466)
Residual standard error: 2.569 on 156 degrees of freedom
Multiple R-squared: 0.7999
Adjusted R-squared: 0.7986

F-statistic: 623.6 on 1 and 156 DF, p-value: < 2.2e-16

	Estimate	Std. Error	t value	Pr(>|t|)
(Intercept)	2.95429	0.49163	6.009	1.27e-08
rich_obs	1.11660	0.04471	24.973	< 2e-16

threshold = 0.75:

Residuals: Min (-4.5176), 1Q (-1.5766), Median (-0.2686), 3Q (1.3165), Max (6.3165)
Residual standard error: 2.102 on 156 degrees of freedom
Multiple R-squared: 0.8489
Adjusted R-squared: 0.8479
F-statistic: 876.3 on 1 and 156 DF, p-value: < 2.2e-16

	Estimate	Std. Error	t value	Pr(>|t|)
(Intercept)	1.85374	1.85374	1.85374	8.39e-06
rich_obs	1.08298	0.03658	29.602	< 2e-16

threshold = 0.85:

Residuals: Min (-3.3522), 1Q (-1.3840), Median (-0.3575), 3Q (1.5577), Max (5.6266)
Residual standard error: 1.729 on 156 degrees of freedom
Multiple R-squared: 0.8785
Adjusted R-squared: 0.8777
F-statistic: 1128 on 1 and 156 DF, p-value: < 2.2e-16

	Estimate	Std. Error	t value	Pr(>|t|)
(Intercept)	1.2674	0.3309	3.83	0.000185
rich_obs	1.0106	0.0301	33.58	< 2e-16

threshold = 0.95:

Residuals: Min (-3.2699), 1Q (-0.9019), Median (-0.1899), 3Q (0.8521), Max (3.7701)
Residual standard error: 1.298 on 156 degrees of freedom
Multiple R-squared: 0.9112
Adjusted R-squared: 0.9106
F-statistic: 1600 on 1 and 156 DF, p-value: < 2.2e-16

	Estimate	Std. Error	t value	Pr(>|t|)
(Intercept)	0.5739	0.5739	2.31	0.0222
rich_obs	0.9040	0.0226	40.01	<2e-16
