# Supplementary material for: Developing custom computer vision models with Njobvu‐AI: A collaborative, user‐friendly platform for ecological research
Source: Ecol Appl. 2025 Sep 11;35(6):e70096. doi: 10.1002/eap.70096 (PMC12426366; doi:10.1002/eap.70096)
Supplement: Supplementary file 4 — Appendix S4. [file EAP-35-e70096-s003.pdf]

APPENDIX S4. Model results for per-image animal counts

Title: Developing custom computer vision models with Njobvu-AI: A collaborative, user-friendly platform for ecological research

Authors: Cara L. Appel, Ashwin Subramanian, Jonathan S. Koning, Marnet Ngosi, Christopher M. Sullivan, Taal Levi, Damon B. Lesmeister

Journal: Ecological Applications

Results summaries from linear models to compare per-image animal counts using confirmed detections (count_true) and model predictions (count_pred) from a YOLOv4 multiclass detector for wildlife species in Nkhotakota Wildlife Reserve, Malawi. Models were run using the *lm* function in Program R.

COUNT MODEL (ADDITIVE):

Call: lm(formula = (count_true – count_pred) ~ -1 + class_true, data = sp_matches_TP)

Residuals: Min (-3.1077), 1Q (-0.1077), Median (-0.1077), 3Q (-0.0067), Max (10.5038)
Residual standard error: 0.47 on 108604 degrees of freedom
Multiple R-squared: 0.08498
Adjusted R-squared: 0.08471
F-statistic: 315.2 on 32 and 108604 DF, p-value: < 2.2e-16

	Estimate	Std. Error	t value	Pr(>|t|)
aardvark	0.00	0.02	0.00	1.00
baboon	0.11	0.00	47.49	0.00
buffalo	0.43	0.02	19.27	0.00
bush_squirrel	0.05	0.07	0.64	0.52
bushbaby	-0.03	0.05	-0.65	0.51

bushbuck	0.01	0.00	1.63	0.10
bushpig	0.06	0.00	13.40	0.00
civet	0.00	0.02	0.10	0.92
domestic_dog	0.11	0.16	0.71	0.48
eland	0.40	0.03	14.27	0.00
elephant	0.14	0.01	25.22	0.00
genet	0.00	0.03	-0.15	0.88
ground_hornbill	0.18	0.03	6.21	0.00
guinea_fowl	0.83	0.03	26.22	0.00
honey_badger	0.05	0.05	0.99	0.32
human	0.06	0.01	5.07	0.00
hyena	0.14	0.13	1.14	0.26
impala	0.14	0.10	1.39	0.16
kudu	0.11	0.01	11.09	0.00
leopard	0.00	0.10	0.00	1.00
mongoose	0.03	0.07	0.34	0.74
porcupine	0.06	0.02	3.61	0.00
reedbuck	0.03	0.02	1.82	0.07
roan	0.00	0.14	0.00	1.00
sable	0.50	0.01	70.41	0.00
scrub_hare	0.00	0.07	0.00	1.00
serval	0.00	0.05	0.00	1.00
small_antelope	0.01	0.00	1.97	0.05
vervet_monkey	0.29	0.02	13.55	0.00
warthog	0.14	0.01	17.00	0.00
waterbuck	0.06	0.01	7.21	0.00
zebra	0.19	0.03	7.44	0.00

COUNT MODEL (PROPORTIONAL):

Call: lm(formula = (count_pred/count_true) ~ -1 + class_true, data = sp_matches_TP)

Residuals: Min (-0.8530), 1Q (0.0015), Median (0.0331), 3Q (0.0331), Max (3.0331)
Residual standard error: 0.1658 on 108604 degrees of freedom
Multiple R-squared: 0.9717
Adjusted R-squared: 0.9717
F-statistic: 1.166e+05 on 32 and 108604 DF, p-value: < 2.2e-16

	Estimate	Std. Error	t value	Pr(>|t|)
aardvark	1.00	0.01	132.53	0.00

baboon	0.97	0.00	1208.78	0.00
buffalo	0.88	0.01	113.03	0.00
bush_squirrel	0.98	0.02	39.09	0.00
bushbaby	1.04	0.02	61.55	0.00
bushbuck	1.00	0.00	573.10	0.00
bushpig	0.98	0.00	598.97	0.00
civet	1.00	0.01	133.62	0.00
domestic_dog	0.94	0.06	17.09	0.00
eland	0.86	0.01	86.47	0.00
elephant	0.95	0.00	491.53	0.00
genet	1.00	0.01	87.80	0.00
ground_hornbill	0.91	0.01	88.62	0.00
guinea_fowl	0.78	0.01	70.45	0.00
honey_badger	0.97	0.02	50.47	0.00
human	1.00	0.00	234.43	0.00
hyena	0.93	0.04	20.95	0.00
impala	0.94	0.04	26.10	0.00
kudu	0.96	0.00	269.43	0.00
leopard	1.00	0.03	28.92	0.00
mongoose	0.99	0.03	37.66	0.00
porcupine	0.97	0.01	169.27	0.00
reedbuck	0.99	0.01	166.66	0.00
roan	1.00	0.05	20.00	0.00
sable	0.89	0.00	357.50	0.00
scrub_hare	1.00	0.02	41.34	0.00
serval	1.00	0.02	53.26	0.00
small_antelope	1.00	0.00	831.60	0.00
vervet_monkey	0.91	0.01	122.38	0.00
warthog	0.96	0.00	339.08	0.00
waterbuck	0.98	0.00	323.04	0.00
zebra	0.93	0.01	102.78	0.00

COUNT MODEL (INTERACTION) – for species classes with max group size > 2:

Call: glm(formula = count_true ~ log(count_pred) * class_true, family = "quasipoisson",
data = sp_matches_TP_gr2)

(Dispersion parameter for quasipoisson family taken to be 0.1326712)
Null deviance: 26316 on 105491 degrees of freedom
Residual deviance: 10172 on 105457 degrees of freedom

	Estimate	Std. Error	t value	Pr(>|t|)
(Intercept)	0.088	0.002	49.102	< 2e-16
log(count_pred)	0.999	0.004	259.579	< 2e-16
class_truebuffalo	0.165	0.016	10.154	< 2e-16
class_truebushbuck	-0.071	0.004	-16.779	< 2e-16
class_truebushpig	-0.030	0.004	-7.394	0.000
class_trueeland	0.226	0.020	11.280	< 2e-16
class_trueelephant	0.032	0.005	6.798	0.000
class_trueground_hornbill	0.102	0.023	4.517	0.000
class_trueguinea_fowl	0.406	0.023	17.575	< 2e-16
class_trueimpala	0.046	0.074	0.616	0.538
class_truekudu	0.012	0.008	1.490	0.136
class_trueporcupine	-0.020	0.013	-1.560	0.119
class_truereedbuck	-0.059	0.013	-4.504	0.000
class_truesable	0.189	0.005	35.470	< 2e-16
class_truesmall_antelope	-0.077	0.003	-24.159	< 2e-16
class_truevervet_monkey	0.133	0.016	8.420	< 2e-16
class_truewarthog	0.015	0.006	2.346	0.019
class_truewaterbuck	-0.027	0.007	-3.912	0.000
class_truezebra	0.046	0.020	2.352	0.019
log(count_pred):class_truebuffalo	0.156	0.030	5.250	0.000
log(count_pred):class_truebushbuck	-0.236	0.025	-9.403	< 2e-16
log(count_pred):class_truebushpig	-0.023	0.009	-2.508	0.012
log(count_pred):class_trueeland	-0.063	0.057	-1.091	0.275
log(count_pred):class_trueelephant	-0.040	0.010	-4.059	0.000
log(count_pred):class_trueground_hornbill	-0.240	0.064	-3.732	0.000
log(count_pred):class_trueguinea_fowl	-0.110	0.034	-3.220	0.001
log(count_pred):class_trueimpala	NA	NA	NA	NA
log(count_pred):class_truekudu	-0.007	0.026	-0.250	0.802
log(count_pred):class_trueporcupine	-0.130	0.045	-2.894	0.004
log(count_pred):class_truereedbuck	0.015	0.074	0.200	0.841
log(count_pred):class_truesable	0.241	0.009	25.352	< 2e-16
log(count_pred):class_truesmall_antelope	-0.143	0.020	-7.092	0.000
log(count_pred):class_truevervet_monkey	-0.017	0.036	-0.491	0.623
log(count_pred):class_truewarthog	0.030	0.014	2.186	0.029
log(count_pred):class_truewaterbuck	-0.040	0.020	-2.028	0.043
log(count_pred):class_truezebra	0.105	0.043	2.421	0.015
